# The Hidden Culprit: Abdominal Tuberculosis Masquerading as Ovarian Cancer in a 22-Year-Old Woman

**DOI:** 10.7759/cureus.95959

**Published:** 2025-11-02

**Authors:** Sara Adam, Saadia Noreldeen, Amira Hassan, Zaid Aldin, Saimah Arif

**Affiliations:** 1 Medicine and Paediatrics, Ipswich General Hospital, Ipswich, GBR; 2 Women and Children's Health, Princess Alexandra Hospital, Harlow, GBR; 3 Obstetrics and Gynaecology, Princess Alexandra Hospital, Harlow, GBR; 4 Radiology, Princess Alexandra Hospital, Harlow, GBR; 5 Histopathology, Princess Alexandra Hospital, Harlow, GBR

**Keywords:** ca 125, computerized tomography, extrapulmonary tuberculosis (ept), tuberculosis (tb), ultrasound scan (us)

## Abstract

Abdominal tuberculosis (TB) is a rare extrapulmonary manifestation of TB in Europe. It may resemble ovarian cancer, especially when ascites, an adnexal mass, and elevated CA-125 levels are present. This case report aims to underline the significance of considering abdominal TB as a differential diagnosis when evaluating ascites with an adnexal mass. The presence of an adnexal mass and ascites should prompt consideration of a TB origin among the possible causes. This case highlights a significant diagnostic challenge, with delays in both diagnosis and management. The patient was evaluated across six specialities (Accident & Emergency, Acute Medicine, Gynaecology, Gynae-Oncology, Infectious Diseases, and Respiratory Medicine), illustrating the complexity and multisystem involvement that contributed to the prolonged diagnostic pathway. Diagnosis of abdominal TB can be challenging and time-consuming. Imaging and tumour markers alone are insufficient. Definitive diagnosis often requires histopathological examination and PCR testing to confirm the presence and nature of the disease.

## Introduction

Tuberculosis (TB) is an infection caused by *Mycobacterium tuberculosis*. It is well known for pulmonary presentation; however, there are also extrapulmonary forms. In 2022, there was an increase in the number of reported cases of TB. There were approximately 170,000 cases of TB in Europe, with extrapulmonary TB accounting for 17% of the cases [1,2]. The increase in reported incidence is likely attributable to the post-COVID-19 recovery in access to and delivery of TB services across many countries, coupled with the implementation of active case-finding initiatives [1].

Abdominal TB is an uncommon form of extrapulmonary TB. However, it is an important differential when treating suspected ovarian malignancy. This is because it can mimic ovarian pathology, as 90% of patients with peritoneal TB may present with ascites [3,4].

The pathophysiology of abdominal TB involves several mechanisms by which *Mycobacterium tuberculosis* reaches the abdominal cavity. The first route is through ingestion of infected materials. The second route is haematogenous dissemination through the bloodstream or lymphatic system from an infected focus elsewhere in the body, such as the lungs. The third route involves direct extension from a nearby infected site, including the fallopian tubes or abdominal lymph nodes [5].

The European Centre for Disease Prevention and Control [1] has estimated that there are over one million TB-related deaths worldwide every year. Patients infected with *Mycobacterium tuberculosis* may be asymptomatic, which is known as latent TB. They will not show symptoms until the infection becomes reactivated. Immunosuppressed people pose a greater risk of developing the disease, with 10% of people with latent TB going on to have active TB secondary to reactivation. The risk is highest in the first two years of infection [6].

## Case presentation

A 22-year-old female university student presented to the acute medical unit with a six-week history of unintentional weight loss of 7 kg, loss of appetite, abdominal pain, bloating, nausea, and vomiting. She was previously fit and well, with a past medical history of asthma managed with salbutamol and steroid inhalers.

Initial blood tests revealed a markedly elevated CA-125 level of 439 U/mL, while all other tumour markers were within normal limits. C-reactive protein remained moderately raised during admission (29-77 mg/L; reference <5 mg/L). A T-Spot test returned positive, indicating a TB-specific immune response (Table 1).

**Table 1:** Initial blood tests on presentation, highlighting an elevated CA-125 and a positive T-Spot result AFP: alpha-fetoprotein; HCG: human chorionic gonadotropin; CEA: carcinoembryonic antigen

Parameter	Result	Reference Range
HCG	<1.2 IU/L	<5 IU/L
AFP	1 U/mL	<10 U/mL
CA-125	439 U/mL	<35 U/mL
CEA	<2 µg/L	<2.5 µg/L
HCG tumour marker	<2 IU/L	<5 IU/L
T-Spot Test		
Neg control spot count	0	0–4
Panel A spot count	>50	0–4
Panel B spot count	>50	0–4
Post control spot count	>20	0–4
Assay result	Positive	-

**Figure 1:**
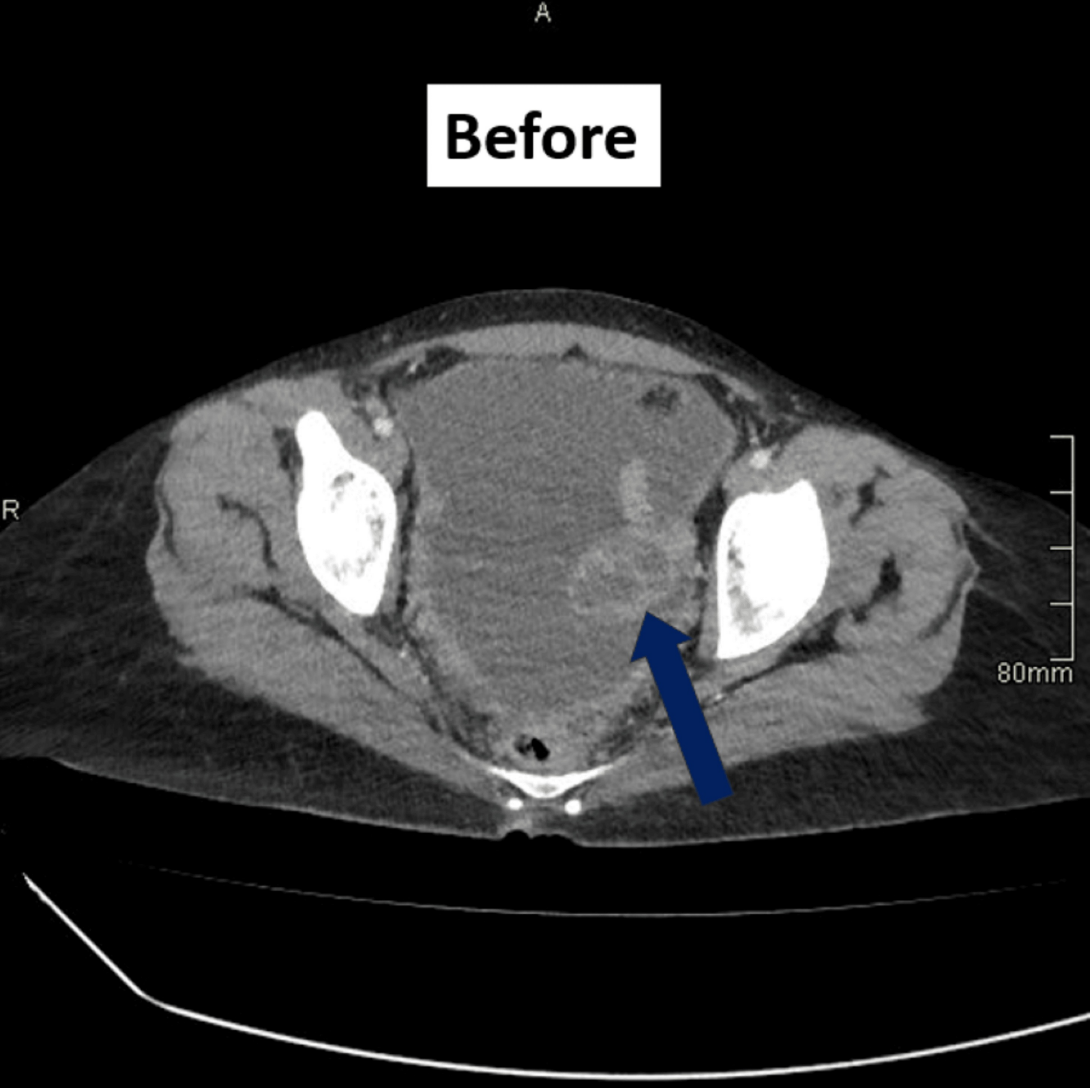
CT scan before treatment. The arrow points to a complex, loculated cystic mass in the left adnexa, present at initial presentation.

**Figure 2:**
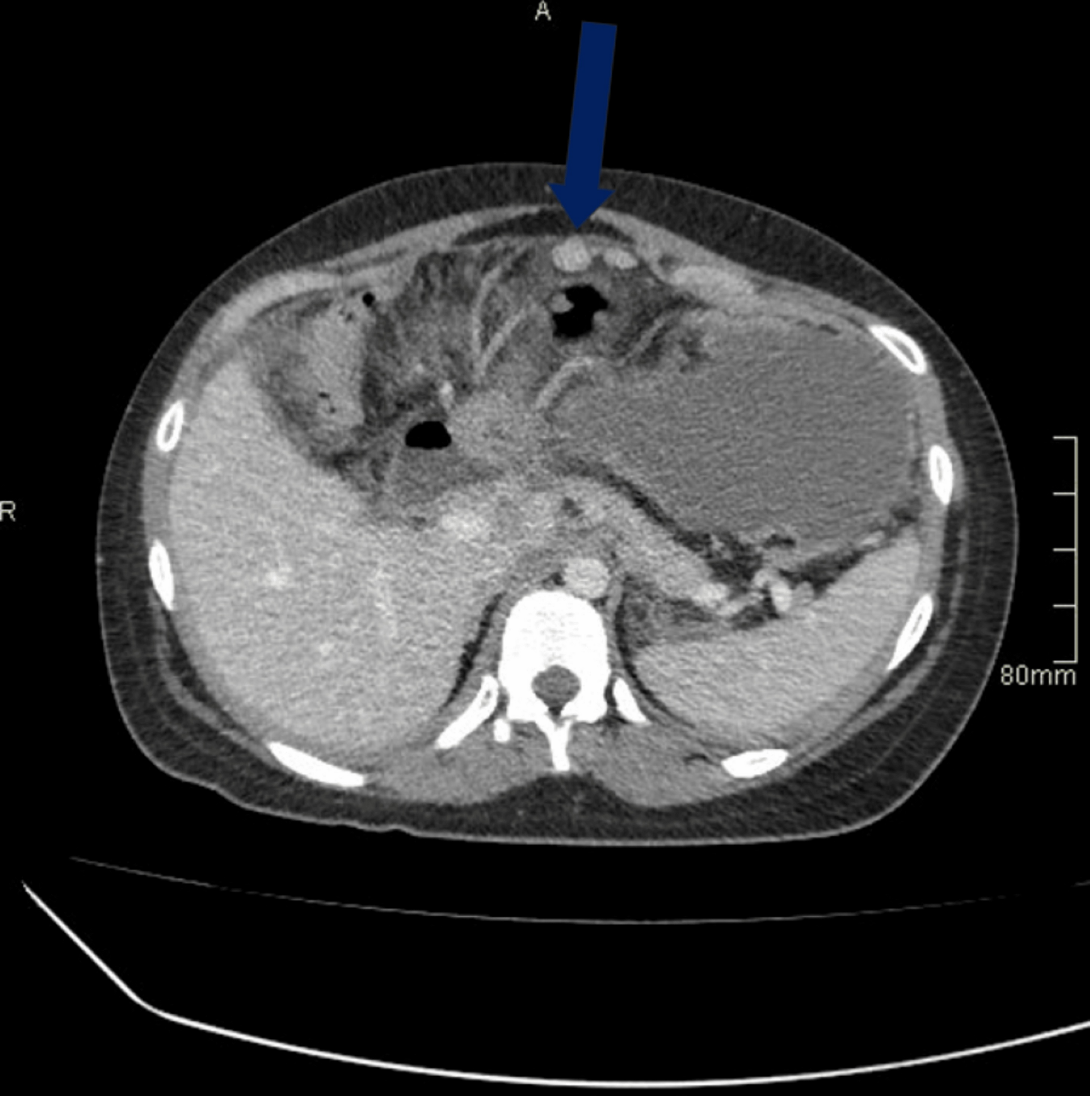
CT abdomen on initial presentation. The arrow points to peri-gastric lymph nodes, which show peritoneal nodularity and ascites, consistent with peritoneal tuberculosis mimicking malignancy.

Computed tomography (CT) of the chest, abdomen, and pelvis demonstrated a complex loculated cystic mass in the left adnexa measuring 58 × 56 × 42 mm (Figure 1), accompanied by pleural effusion, large-volume ascites, peritoneal deposits, and small-bowel thickening. There was widespread lymphadenopathy, including mesenteric, para-aortic, mediastinal, and retrocaval nodes, and several soft-tissue nodules in the peri-gastric and left subhepatic regions, likely representing lymph nodes (Figure 2). These findings were initially interpreted as consistent with advanced ovarian carcinoma, and the patient was transferred to the gynaecology-oncology ward. A chest drain was inserted to manage the pleural effusion, and she and her family were counselled regarding the provisional diagnosis and offered emotional and psychological support.

Ultrasound-guided drainage of the ascitic fluid was performed, and the sample was submitted for cytological and microbiological analysis. Cytology revealed a small number of lymphocytes and macrophages but no malignant cells. Immunohistochemistry confirmed granulomatous inflammation. In light of these unexpected findings, a detailed family and travel history was revisited. The patient had been born and raised in the United Kingdom but had travelled five months earlier to a rural area of Pakistan, her parents' country of origin, where she stayed for three months. She reported no known contact with individuals exhibiting symptoms of TB and had received childhood BCG (Bacillus Calmette-Guérin) vaccination.

Given the granulomatous findings, peritoneal tissue and ascitic fluid samples were examined for mycobacteria and fungi. Ziehl-Neelsen (ZN), Grocott, and PASD (periodic acid-Schiff with diastase) stains were negative; however, this did not exclude TB as a possible cause. An omental biopsy showed fibroadipose tissue containing multiple non-caseating epithelioid granulomas without foreign material or evidence of malignancy. The differential diagnosis included infectious causes (mycobacterial, fungal, and parasitic) and non-infectious conditions such as sarcoidosis, Crohn's disease, endometriosis, and foreign-body reactions.

Upper gastrointestinal endoscopy (OGD) with gastric and duodenal biopsies was carried out to investigate other granulomatous or infiltrative disorders. Gastric biopsies revealed moderate chronic inflammation with foveolar hyperplasia and *Helicobacter pylori* organisms, consistent with reactive gastropathy. Duodenal biopsies showed mild intraepithelial lymphocytosis with preserved villous architecture. There was no evidence of dysplasia, malignancy, or granulomatous disease in either site.

Taking into account the positive T-Spot test, histological evidence of non-caseating granulomas, compatible radiological findings, and supportive travel history, a diagnosis of abdominal TB was established after multidisciplinary discussion involving infectious-disease, respiratory, and gynaecology teams.

The patient was commenced on standard anti-tubercular therapy, consisting of Rifater (six tablets once daily), Ethambutol 1000 mg once daily, Pyridoxine 10 mg once daily, and Metoclopramide 10 mg three times daily for symptomatic control. She tolerated the regimen well and experienced no significant adverse effects.

**Figure 3:**
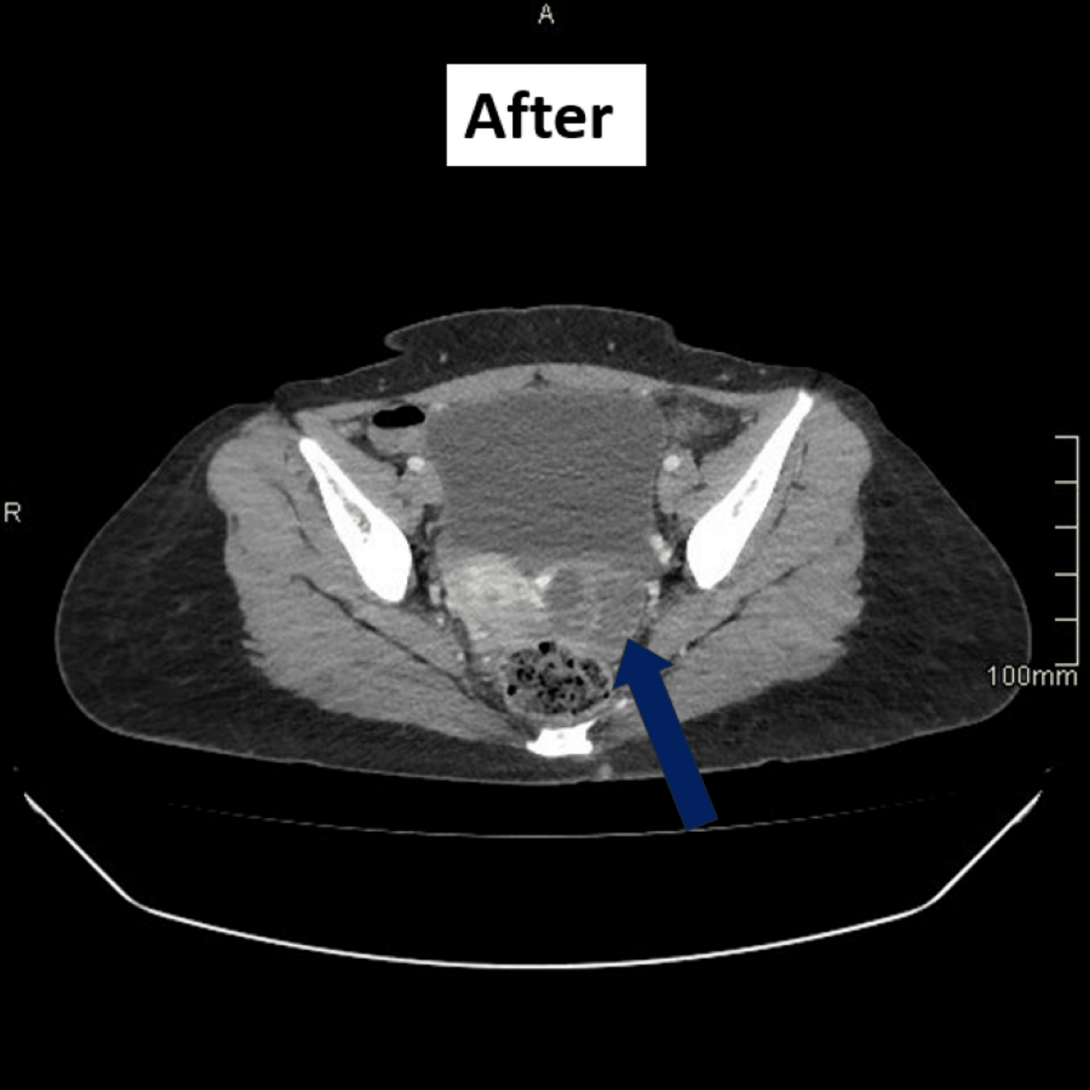
CT after initial treatment. The arrow points to the left adnexal mass, which has reduced in size after three months of treatment.

After three months of therapy, the patient reported significant improvement in appetite, weight gain, and resolution of abdominal discomfort. Follow-up CT imaging demonstrated a reduction in mediastinal lymphadenopathy and ascitic volume, along with shrinkage of the left adnexal mass (Figure 3). Overall appearances were consistent with a favourable treatment response. She remains under ongoing review in the infectious-disease and respiratory clinics, with continuation of anti-tubercular therapy for at least six months.

## Discussion

This case highlights the variable manifestations of extrapulmonary tuberculosis (EPTB). Often, EPTB may present either as an isolated condition or in combination with pulmonary involvement. The emergence of human immunodeficiency virus (HIV) and acquired immunodeficiency syndrome (AIDS) has significantly altered the epidemiology of TB, bringing renewed attention to EPTB. This form of TB can affect virtually any organ system in the body. Due to its atypical clinical manifestations, obtaining tissue samples for definitive diagnosis can be challenging, and traditional diagnostic methods, such as the ZN stain, often yield inconclusive results, leading to delays in diagnosis. However, advancements in imaging techniques, such as CT, magnetic resonance imaging (MRI), laparoscopy, and endoscopy, have greatly improved the anatomical localisation of EPTB. Fortunately, the disease generally responds well to standard anti-TB therapy [7-9].

This case has served as a valuable learning experience for all the clinicians involved. The delay in establishing a diagnosis was primarily due to a low index of suspicion for EPTB, particularly abdominal TB. Most of the healthcare professionals managing the case had limited prior exposure to EPTB, which contributed to diagnostic uncertainty and delays.

Clinically, the patient's presentation closely mimicked that of advanced ovarian carcinoma, leading to an initial focus on malignancy. Unfortunately, inflammatory markers were not adequately correlated with imaging findings to support a broader differential diagnosis. Another contributing factor was the omission of a detailed travel history, which could have highlighted potential exposure risks in endemic regions.

Additionally, ascitic fluid was submitted for cytological examination and staining only, without biochemical analysis. The omission of adenosine deaminase (ADA) testing represents a missed diagnostic opportunity, as elevated ADA levels (>30-40 IU/L) are considered a crucial marker in identifying TB ascites and could have significantly supported the diagnosis.

Furthermore, the patient's history of childhood BCG vaccination was incorrectly assumed to confer complete protection against TB, further reducing clinical suspicion.

In this case, histological examination revealed non-caseating granulomatous inflammation without caseous necrosis or demonstrable acid-fast bacilli. While caseation is traditionally associated with TB, its absence does not exclude the disease, particularly in extrapulmonary forms. Several studies have shown that non-caseating granulomas may be present in up to one-third of EPTB cases [10-12]. The paucibacillary nature of extrapulmonary infection also means ZN staining and even cultures are frequently negative. Thus, diagnosis often relies on a composite approach that integrates clinical features, radiological findings, immunological tests, and therapeutic response [10,13]. In this patient, the recent travel to a TB-endemic area, characteristic radiological findings, a positive interferon-gamma release assay (T-Spot test), and subsequent clinical and radiological improvement on anti-tubercular therapy together provided sufficient evidence to establish the diagnosis.

The main differential diagnosis was sarcoidosis, as it was also characterised by non-caseating granulomas [11]. However, several features argued against a diagnosis of sarcoidosis. The disease most often affects the lungs, skin, and lymph nodes, with isolated peritoneal involvement being extremely rare. Classic features such as bilateral hilar lymphadenopathy, pulmonary infiltrates, and hypercalcaemia were absent in this patient. Furthermore, the positive T-Spot result strongly supported TB infection, as interferon-gamma release assays do not typically yield positive results in sarcoidosis [12,13]. The marked symptomatic and radiological improvement with anti-tubercular therapy also made sarcoidosis and other non-infectious causes (e.g., Crohn's disease, endometriosis-related granulomas, or foreign-body reaction) highly unlikely.

This case reinforces the importance of maintaining a broad differential diagnosis, especially in atypical presentations, and highlights the need for increased awareness and training in recognising extrapulmonary manifestations of TB. It also highlights the diagnostic value of combining clinical context, imaging, immunological testing, and therapeutic responses rather than overrelying on histology alone when establishing the diagnosis of extrapulmonary tuberculosis.

## Conclusions

Abdominal TB is a rare form of EPTB. Its presentation often mimics disseminated cancer, making diagnosis challenging, particularly in non-endemic regions. This case highlights the importance of comprehensive history-taking and maintaining a broad differential diagnosis when evaluating patients with nonspecific abdominal symptoms, particularly when initial findings suggest malignancy. Additionally, an elevated CA-125 level should be interpreted with caution and correlated with other inflammatory markers. It should not be automatically considered indicative of malignancy, as CA-125 can also be raised in various benign conditions, including infections, endometriosis, and other inflammatory states.

With evolving patterns of EPTB caused by factors such as increased global travel, migration, and the rising prevalence of immunosuppressive conditions like HIV, clinicians must have a high index of suspicion for EPTB. EPTB has different clinical presentations that often mimic other diseases. This makes early diagnosis vital for early treatment and improved patient outcomes.
